# The incorporation of emotion-regulation skills into couple- and family-based treatments for post-traumatic stress disorder

**DOI:** 10.1186/s40779-017-0130-9

**Published:** 2017-06-30

**Authors:** Deborah A. Perlick, Frederic J. Sautter, Julia J. Becker-Cretu, Danielle Schultz, Savannah C. Grier, Alexander V. Libin, Manon Maitland Schladen, Shirley M. Glynn

**Affiliations:** 1 JJPeters Department of Veterans Affairs Medical Center and VISN2 South Mental Illness Research, Education and Clinical Center, 130 West Kingsbridge Rd, Bronx, NY 10468 USA; 2 Department of Psychiatry, Icahn School of Medicine at Mount Sinai, 1 Gustave L. Levy Pl, New York, NY 10029 USA; 3 Southeast Louisiana Veterans Health Care System, 1601 Perdido St, New Orleans, LA 70112 USA; 4 Tulane University School of Medicine, 1430 Tulane Ave, New Orleans, LA 70112 USA; 5 Research and Development, Washington DC VA Medical Center, 50 Irving St NW, Washington, DC 20422 USA; 6 Georgetown University Medical Center, 3800 Reservoir Rd NW, Washington, DC 20007 USA; 7 VA Greater Los Angeles Healthcare System, 11301 Wilshire Blvd, Los Angeles, CA 90073 USA; 8 David Geffen School of Medicine, UCLA, 10833 LeConte Ave #12138, Los Angeles, CA 90095 USA

**Keywords:** couples, family, post-traumatic stress disorder, emotional regulation

## Abstract

Post-traumatic stress disorder (PTSD) is a disabling, potentially chronic disorder that is characterized by re-experience and hyperarousal symptoms as well as the avoidance of trauma-related stimuli. The distress experienced by many veterans of the Vietnam War and their partners prompted a strong interest in developing conjoint interventions that could both alleviate the core symptoms of PTSD and strengthen family bonds. We review the evolution of and evidence base for conjoint PTSD treatments from the Vietnam era through the post-911 era. Our review is particularly focused on the use of treatment strategies that are designed to address the emotions that are generated by the core symptoms of the disorder to reduce their adverse impact on veterans, their partners and the relationship. We present a rationale and evidence to support the direct incorporation of emotion-regulation skills training into conjoint interventions for PTSD. We begin by reviewing emerging evidence suggesting that high levels of emotion dysregulation are characteristic of and predict the severity of both PTSD symptoms and the level of interpersonal/marital difficulties reported by veterans with PTSD and their family members. In doing so, we present a compelling rationale for the inclusion of formal skills training in emotional regulation in couple−/family-based PTSD treatments. We further argue that increased exposure to trauma-related memories and emotions in treatments based on learning theory requires veterans and their partners to learn to manage the uncomfortable emotions that they previously avoided. Conjoint treatments that were developed in the last 30 years all acknowledge the importance of emotions in PTSD but vary widely in their relative emphasis on helping participants to acquire strategies to modulate them compared to other therapeutic tasks such as learning about the disorder or disclosing the trauma to a loved one. We conclude our review by describing two recent innovative treatments for PTSD that incorporate a special emphasis on emotion-regulation skills training in the dyadic context: structured approach therapy (SAT) and multi-family group for military couples (MFG-MC). Although the incorporation of emotion-regulation skills into conjoint PTSD therapies appears promising, replication and comparison to cognitive-behavioral approaches is needed to refine our understanding of which symptoms and veterans might be more responsive to one approach versus others.

## Background

Post-traumatic stress disorder (PTSD) is a potentially chronic, impairing disorder that is characterized by re-experience and hyperarousal symptoms as well as negative cognitions and avoidance of trauma-related stimuli [1]. In returning veterans, PTSD frequently presents with co-occurring depression, substance abuse and traumatic brain injury [2]. Although PTSD is an individually diagnosed disorder, many of its core symptoms can lead to disruptions in close relationship such as detachment or estrangement or have the potential to create interpersonal conflict due to irritability, anger, severe agitation [3] or reckless behavior [4–7]. In this paper, we discuss the important role that emotion regulation, defined as the ability to change the frequency, intensity, and/or duration of emotion [8], plays in the process of veterans’ learning to join with their partner or family member to reduce veterans’ PTSD and its negative impact on the veterans’ intimate relationships. We begin by reviewing emerging evidence that: 1) high levels of emotion dysregulation are characteristic of and predict PTSD severity and 2) high levels of emotion dysregulation are associated with the severity of interpersonal and/or marital difficulties among veterans with PTSD and their partners or family members. Second, we discuss both the rationale and the therapeutic strategies for incorporating emotion-regulation skills training into couple- and family-based interventions for PTSD. Third, we review the evolution of couple therapy for veterans with PTSD, particularly focusing on randomized clinical trials that were conducted with veterans. In this context, we describe two recent innovative couple-based treatments for PTSD that incorporate a special emphasis on emotion-regulation skills training in the dyadic context. Structured approach therapy (SAT) [9] seeks to improve couples’ ability to manage trauma-related emotions by providing skills training in awareness, labeling, and acceptance of emotions as well as in distress tolerance. Multi-family group for military couples (MFG-MC) [10, 11] teaches skills in mindfulness, distress tolerance, and more advanced emotion-regulation strategies to add this important dimension to communication skills training in subsequent sessions. Fourth and finally, we discuss the limitations and challenges of the work to date and future directions for research in this area.

## PTSD symptoms, emotion dysregulation, and family/marital difficulties

Many returning veterans with PTSD show emotion regulation problems [12–14], and difficulties in emotion regulation have been linked to PTSD symptom severity [15, 16]. Such difficulties include problems in the identification and expression of emotion as well as in the ability to tolerate negative affect and traumatic event cues without feeling overwhelmed or losing control. Specific problems with emotion regulation have been differentially associated with the severity of PTSD symptom clusters. For example, Monson et al. [14] found that difficulty associated with describing feelings was a significant predictor of the level of re-experience symptoms only, whereas negative affect was associated with the severity of avoidance/numbing, hyperarousal and re-experience symptoms among veterans who were enrolled in an intensive PTSD treatment program. Anger is also predictive of PTSD severity, particularly hyperarousal symptoms [17]. Nevertheless, a survey of 676 veterans [18] found that self-reported aggressive urges were associated with the severity of re-experience symptoms, whereas difficulty managing anger was associated with the severity of avoidance symptoms. Studies of combat veterans have consistently found significant associations between the PTSD symptom level and anger, even after accounting for demographic and exposure variables [19].

Not surprisingly, high levels of PTSD have frequently been associated with relationship distress (e.g., [20–22]), poor family functioning in veterans [2, 23] and intimate partner violence in veterans [24]. Taft and colleagues [24] found medium-sized associations in a meta-analytic investigation of 31 studies on the association between PTSD severity and interpersonal psychological and physical aggression, with the largest effects observed in military samples. Evans et al. [2] evaluated the impact of PTSD symptom clusters on family functioning via path analysis, finding both a significant direct effect of avoidance symptoms on overall family functioning and an indirect path via the effects of avoidance symptoms on depression. Hyperarousal symptoms had an indirect association with family functioning that was mediated by the association between arousal symptoms and anger, whereas re-experience symptoms did not impact family functioning in this study.

## Rationale and strategies for incorporating emotion-regulation skills training into couple- and family-based interventions for PTSD

The association between PTSD symptom severity and both emotion dysregulation and couple and family relationship distress makes a compelling case for incorporating emotion-regulation skills into family-based interventions for PTSD. Learning theories of PTSD predict that the increased exposure to trauma-related memories and emotions will decrease the veterans’ PTSD. However, successful exposure requires the veterans and their partners to develop the ability to manage the uncomfortable emotions that they previously avoided. Studies have shown that adapting to PTSD-related emotions requires veterans to develop the ability to increase their acceptance and awareness of aversive emotions while also accessing effective emotion-regulation strategies and minimizing impulsivity and avoidance [25]. Learning and practicing emotion-regulation skills has the potential to be particularly powerful in the dyadic context for returning Operation Enduring Freedom/Operation Iraqi Freedom/Operation New Dawn (OEF/OIF/OND) veterans. The majority of OEF/OIF/OND couples is married/cohabiting and faces major challenges in association with reconnecting and renegotiating roles post-deployment [26]. Couples’ interactions often elicit strong emotions, which can lead to behaviors that create stress and can lead to relationship dissolution if the partners’ emotion-regulation skills are poor. One report found that 35% of veterans who were receiving Veterans Affairs (VA) care reported separation or divorce within 3 years of their homecoming [27]. Couples who learn to regulate emotions successfully through conjoint work for PTSD might experience the simultaneous benefit of enhancing the relationship while addressing the disorder.

## Evolution of couple- and family-based interventions for PTSD

Several papers on conjoint or family-based approaches to the treatment of combat-related PTSD have been published in the last 30 years. These interventions often not only incorporate components that have been found to be effective in individual treatments (e.g., cognitive restructuring) but also include interventions that involve dyadic work such as communication skills training. Although the developers of these interventions all acknowledge the importance of emotions in PTSD, the treatments vary widely in their relative emphasis on helping participants to acquire strategies to modulate them compared to other therapeutic tasks such as learning about the disorder or disclosing the trauma to a loved one. None of the interventions is defined as primarily involving emotion regulation, although teaching skills such as active listening and taking a time-out clearly promotes more control over affect. In this section, a brief overview of couples work with PTSD is presented, with an emphasis on veteran samples. We begin with preliminary papers, which often provided theoretical applications and case descriptions of established couples interventions to combat-related PTSD. We then move to presentations of more rigorously controlled trials of conjoint interventions with veterans. We conclude with more detailed descriptions of SAT and MFG-MC [9–11], two newer veteran couples interventions for PTSD that have an explicit focus on emotion regulation.

### Preliminary work: uncontrolled trials and case studies

The distress that is experienced by many veterans of the Vietnam War and their partners prompted a strong interest in developing conjoint interventions that could both alleviate symptoms of PTSD and strengthen family bonds. Many clinicians wrote thoughtfully about the difficulties associated with family reintegration after combat and began to develop intervention models that were typically grounded in existing structural, strategic, narrative and/or dynamic approaches to family therapy to facilitate the recovery of the traumatized veteran and the development of a new family equilibrium [28–34]. These authors often illustrated their approaches with compelling clinical vignettes but did not publish empirical data to support their models. Consistent with the family therapy traditions from which they evolved, these approaches were primarily experiential and included little formal skills training.

The late 1980s and early 1990s ushered in a new era in couples interventions, with a greater emphasis on methodological rigor and empirical testing of outcomes. Although these new conjoint interventions were first conceptualized as a way to address relationship distress, they have subsequently been applied to PTSD. Johnson et al.’s [35] emotionally focused couple therapy (EFT) is grounded in attachment theory and proposes that repairing attachment ruptures and restoring intimate connections are the key therapeutic tasks in couple therapy. EFT consists of three stages: de-escalation of the couple’s negative cycle (stage I), restructuring of problematic interactions (stage II), and consolidation/integration (stage III). In successive steps in stage II, individuals are assisted in voicing both their attachment needs and their deep emotions and then prompted to express acceptance and compassion for their partner’s attachment needs and emotions. Over time, as trust develops between the partners, increasingly more conflictual topics are addressed. Interactions are guided by the therapist, who has an overarching goal of supporting the (re)attachment of the partners. However, the attention paid here to understanding and modulating emotions in the service of securing this connection could also be understood in the rubric of emotion regulation.

EFT has been evaluated in distressed couples in the community in open as well as randomized controlled trials (RCTs), with relatively consistent findings of improvements in relationship satisfaction and/or empathy resulting from engagement in therapy ([36–38]; see [39] for a review). There have also been EFT investigations in couples facing the aftermath of trauma. Improvements in both the relationship and trauma symptoms were observed in 10 couples who were participating in EFT in which one member had a history of childhood sexual abuse and a diagnosis of PTSD [40]. Dalton et al. [41] conducted a randomized controlled trial to examine the efficacy of EFT in 32 couples in which the female partner had experienced past childhood abuse. A diagnosis of PTSD was not an inclusion requirement. The couples were randomly assigned to 24 sessions of EFT or a waitlist control group. Compared to the waitlist condition, participation in EFT was associated with significantly greater relationship satisfaction scores posttreatment, although there was no impact of EFT on trauma symptoms. As cited in Wiebe and Johnson [39], Weissman et al. conducted an open EFT trial with 7 veterans who were diagnosed with PTSD and found reductions in PTSD symptoms as well as increases in mood and relationship satisfaction. Greenman and Johnson [42] also applied the EFT model to PTSD treatment in veterans using a case example. Outcome data were not available, as the couple was still in treatment when the article was written. Unfortunately, more rigorous research of EFT with combat veterans is lacking to date.

Erbes, Polusny, MacDermid, and Compton [43] applied integrative behavioral couple therapy (IBCT; [44]) to treat combat-related PTSD. The goal of IBCT is to reduce marital distress by enhancing partners’ acceptance of each other. The intervention entails providing initial tailored feedback to the couple based on a thorough assessment, promoting partners’ empathy towards each other and supporting the couples’ adoption of a unified approach to the problems that they face, rather than blaming each other. Some couples are provided with advanced work relating to distress tolerance in which they are guided to interact in the session around a previously emotionally loaded issue using their new empathy and unified approach to the problem. Erbes et al. [43] posited that IBCT might be particularly effective for PTSD survivors because it reduces couple conflict and increases intimacy through fostering acceptance, tolerance, and the expression of primary emotions such as fear or sadness that often underlie the chronic anger that is associated with PTSD. However, aside from the limited work on distress tolerance highlighted above, the approach does not involve any formal emotion-regulation skills training. Although there is a considerable evidence base for IBCT in community samples [45], it has not been evaluated in controlled research for the treatment of PTSD. The application of IBCT to PTSD in Erbes et al. [43] has been illustrated with only a case example to date.

Sherman and colleagues developed a conjoint education and support program Reaching Out To Educate and Assist Caring, Healthy (REACH) Families [46] tailored to the unique needs of families of returning OEF/OIF/OND veterans that incorporated aspects of the multiple-family group therapy format for serious mental illness (SMI) proposed by McFarlane et al. [47]. REACH is primarily educational but includes some discussion of managing negative affect as well as formal skills training and out-of-session practice. Sherman and colleagues have not tested the benefits of REACH in randomized trials but have presented data on knowledge gains accrued in the groups and participant satisfaction [46] that suggest that participants learn about PTSD and other mental health issues and find the intervention to be accessible and helpful.

### Larger randomized clinical trials of couple/family work relating to PTSD

Researches in mental health in the late 1980s and 1990s were influenced by growing specification regarding the impact of environmental stressors, including family tension and conflict, on outcomes relating to psychiatric disorders. The diathesis-stress model [48] proposes that the extent of the expression of a biological vulnerability to a disorder (i.e., the diathesis) is influenced by the degree of exposure to stress. As applied to PTSD, the theory proposes that once the disorder develops (as a result of exposure to extreme environmental stress), the survivor is extremely sensitive to subsequent ambient stress, including negative appraisals by relatives. This theoretical framework implies that potentially effective interventions might focus on the reduction of ambient stress by teaching the trauma survivor and his/her loved ones specific skills to promote effective communication and problem solving to minimize conflict in the home environment and cope with life’s challenges successfully.

Behavioral family therapy (BFT) is grounded in the diathesis-stress model of psychiatric illness and includes illness education, communication skills training, and problem-solving instruction. Glynn et al. [49] conducted a randomized trial comparing the additive benefits of BFT to prolonged exposure in a trial of Vietnam veterans diagnosed with combat-related PTSD. Vietnam veterans and a family member (90% of whom were conjugal partners) were randomized to a) wait list, (b) 18 sessions of twice-weekly exposure therapy (ET), or (c) 18 sessions of twice-weekly exposure therapy followed by 16 sessions of behavioral family therapy (ET + BFT). Although the study findings did not support the hypothesis that adding BFT to ET would improve treatment outcomes, they did indicate that both the ET and the ET + BFT conditions improved re-experience and hyperarousal symptoms compared to the wait list control group. Although they were not statistically significant, the ET + BFT group was associated with reductions in re-experience and hyperarousal symptoms that were approximately twice the magnitude of those obtained in the ET group. Additionally, there was an overall effect size advantage (*d* = 0.46) for ET + BFT compared to ET alone. There was no effect on numbing or avoidance symptoms. This pattern of results suggested that family interventions might have some value in treating PTSD. However, more interventions need to be developed.

Monson et al. [50] developed a manualized conjoint, skills-focused treatment for PTSD called cognitive-behavioral conjoint therapy (CBCT). CBCT for PTSD consists of 15 75-min sessions and incorporates many aspects of cognitive processing therapy [51, 52] conducted in a conjoint frame. As such, the primary therapeutic goal is to harness social support to modify dysfunctional trauma-related cognitions to reduce PTSD and support successful reintegration. CBCT has three phases: (1) education about PTSD and its effect on relationships and safety building, (2) communication skills training and couple-oriented in vivo exposure to overcome behavioral and experiential avoidance, and (3) cognitive interventions aimed at changing problematic trauma appraisals and beliefs that maintain PTSD and relationship problems. A key therapeutic goal is to support the dyadic frame. That is, the couple engages in the healing activities together and shares responsibility for recovery. There have been positive findings from small uncontrolled studies with combat veterans who were diagnosed with PTSD [53, 54]. The RCT confirming the benefits of CBCT on PTSD symptoms (effect size =1.13 on the Clinician-Administered PTSD Scale [55]) and relationship functioning (effect size = 0.47 for the survivor on the Dyadic Adjustment Scale [56]) was conducted with a mixed community veteran sample with broad trauma exposure. There were 9 veteran participants, 2 of whom had a combat-related PTSD diagnosis.

### Newer couples treatments for PTSD with an emphasis on emotion-regulation skills training

Although the interventions described above incorporate some features that are designed to address emotion dysregulation in association with PTSD and the negative impact on couples, they have not systematically implemented emotion-regulation skills training as explicit therapeutic tasks. These studies do not provide guidelines for defining which emotion-regulation skills should be included and which symptoms or deficits are most likely to be addressed. Because emotion regulation might be crucial to achieving favorable PTSD treatment outcomes [54], it is important to base our interventions on theoretical models of emotions and emotional functioning that are consistent with our understanding of PTSD [12, 53]. It has been hypothesized that the experience of trauma generates acute reactions of fear and anxiety, followed by the development of more enduring emotions that require regulation across varied environmental and social contexts [8, 57]. The processing and regulation of emotions have been described as a set of experiential, physiological, and behavioral responses that persist over time as an individual learns first to experience and tolerate the generation of internal “core affects” [57]) and then to learn strategies to modulate these emotions within the context of environmental challenges and internally generated goals and cognitions [58]. Conceptual models that differentiate between the generative and the regulatory aspects of emotional control [12, 58] are consistent with data showing that different neural systems mediate the relationship between fear-related emotional reactivity and emotional inhibition and control [58, 59]. Similarly, the behavioral responses to sudden increases in trauma-related emotions [60] are distinctly different from the more complex emotional states that develop in people who must adapt to trauma and adversity over more extended periods of time [61].

The newer PTSD couple interventions that are presented next are grounded in this conceptualization of emotion. They incorporate explicit strategies to increase distress tolerance and emotion-regulation skills while enhancing the couple’s awareness and understanding of affect. Complementary therapeutic goals include engendering acceptance of emotions and the ability to regulate behaviors in accordance with long-term relationship goals, even while experiencing strong negative emotions. This training in the acceptance and regulation of emotions allows the veteran and his or her partner to use situationally appropriate emotion-regulation strategies in a flexible manner to modulate emotional responses [62, 63]. We have developed treatment models for both individual (SAT [9]) and couple group interventions (MFG-MC) [11] that incorporate emotion-regulation (ER) skills training as a major therapeutic component to treat PTSD with combat veterans and have had some success.

### Structured therapy approach

Data from Glynn et al.’s [49] study described above showing that BFT + ET reduced re-experience and hyperarousal symptoms but not symptoms of avoidance and emotional numbing indicated the need to target the latter symptoms more directly. Sautter and Glynn used these findings as the basis for a new couple-based PTSD treatment called structured approach therapy (SAT). Conducted by a single therapist with a single couple, SAT is designed to help the partners to decrease their avoidance of trauma-related stimuli and to enhance their emotion regulation.

SAT is a phasic PTSD treatment that includes out-of-session practice. The first phase of SAT consists of conjoint illness education that provides the couple with information regarding trauma and describes how trauma impacts the processing of emotions that are crucial for maintaining intimate relationships. The second phase of SAT consists of a skills-training component in which the partners are taught to identify, label and communicate about their avoidance of trauma-related stimuli. They are simultaneously provided with emotion-regulation tools to cope with trauma-related emotions, rather than engage in the avoidance that perpetuates PTSD. More specifically, they learn skills to activate positive emotions and engage in couple soothing and empathic mutual support that increases distress tolerance [64]. For example, couple soothing exercises help couples to identify and engage in behaviors to cope with negative affect by promoting feelings of relaxation and intimacy. These soothing behaviors can include traditional relaxation techniques such as deep breathing, positive thinking, or imagining a relaxing place as well as activities that they enjoy doing together such as cooking or exercising. This process of teaching couples to decrease emotional avoidance while increasing support for disclosing and discussing traumatic memories and emotions reduces veterans’ vulnerability to PTSD while increasing couples’ psychological resilience.

The couples then participate in 6 disclosure-based exposure sessions in which the veterans are prompted to reveal and discuss trauma-related memories and emotions with their partners. This disclosure process is intended to expose the veterans gradually to trauma-related emotions. Couples learn to approach and not avoid the trauma-related problems that have devastated their relationship in the past. Through this conjoint SAT, the veteran has multiple trials of exposure to trauma-related memories and emotions to habituate to anxiety cues while also cognitively processing the trauma in a supportive context.

SAT’s emphasis on disclosure is grounded in findings that returning veterans who speak about their combat trauma to an intimate partner experience decreases in posttraumatic stress [65] while simultaneously improving their relationship quality [66]. It is important to emphasize that SAT does *not* involve exposing the veteran to the same intensity of trauma-related emotions as prolonged exposure. Instead, SAT is designed to permit opportunities for anxiety habituation during treatment while also providing instruction on the communication, emotion regulation, and anxiety-management skills that allow the couple to use disclosure practices to confront avoidance trauma both when they engage in disclosure work in the last 6 sessions and after the conclusion of treatment. For example, skills training in acceptance allows them to tolerate challenging emotions more effectively as the veteran discloses his or her traumatic experiences. The couple is also coached to use their empathic communication skills to identify and discuss their emotional responses to the disclosure. For instance, the veteran’s partner is coached to validate the veteran’s trauma-related emotions and encourage him or her to join in a couple-soothing exercise designed to provide comfort while discussing the emotional challenges of confronting the trauma. Incorporating emotion-regulation and communication skills into the disclosure phase allows the couple to process traumatic memories and emotions in an accepting and supportive dyadic context.

#### Efficacy of Structured Approach Therapy

The initial 12-session manual-based treatment was tested in an uncontrolled trial with Vietnam veterans with PTSD and their spouses. Participating veterans showed significant reductions in avoidance and numbing symptoms in addition to significant decreases in their overall PTSD scores [9]. Based on these positive findings, the manual was modified to meet the needs of post-911 veterans [67] and evaluated in an open trial with seven Iraq and Afghanistan veterans and their partners and, more recently, in a randomized clinical trial comparing a 12-session SAT intervention with a 12-session couple-based education condition called PTSD family education (PFE) [68]. Seventy-six percent of the 57 OEF/OIF/OND couples who were randomly assigned to a group were retained through three months of follow-up assessments. Intent-to-treat analysis revealed that both the SAT and the PFE veteran groups showed significant reductions in self-reported and clinician-rated PTSD during the treatment period and at 3-months follow-up. However, the veterans who were randomly assigned to SAT showed significantly greater reductions in PTSD than those who were randomly assigned to PFE. Specifically, every couple who received SAT had a reduction in veteran PTSD within just twelve sessions, which was maintained over a 3-months follow-up period. Fifteen of the 29 (52%) veterans in SAT and two out of the 28 (7%) veterans in PFE no longer met the DSM-IV-R criteria for PTSD (operationalized as exceeding a total CAPS score of 45) at 3-months follow-up. Additional analyses revealed that the veterans’ decreases in fear of intense emotions (emotion generation) and their improved emotion-regulation skills partially mediated the relationship between treatment with SAT vs. PFE and reductions in PTSD symptoms (CAPS change score ĉ = 1.03, *P* = .003). These data indicate that improving emotion regulation is an important element in the successful treatment of PTSD with SAT [69].

### Multi-family group for military couples (MFG-MC)

Although individual couples treatment is often used with PTSD, group treatments have the advantage of permitting participants to learn from each other and can also reduce stigma. They are also more efficient. Multi-family group (MFG) for military couples with trauma associated with combat stress/exposure and/or mild traumatic brain injury (mTBI) is an adaptation of multi-family group treatment, an evidence-based treatment for serious mental illness that uses education, problem-solving skills training and support to reduce symptoms and improve functional outcomes [47]. Perlick and colleagues adapted the MFG approach to address the needs of post-911 veterans with mTBI and/or full or sub-syndromal PTSD in an open, feasibility trial [10, 11]. They are currently evaluating this treatment in an ongoing VA-funded multi-site RCT comparing the benefits of MFG-MC compared to health education (HE).

The MFG-MC model uses a structured, behavioral approach to provide veterans and their partners with education and problem-solving instruction as well as emotion-regulation and communication skills training to improve coping and help couples to reconnect through positive behavioral exchanges. MFG-MC consists of three sequential components: 1) “joining” in which clinicians meet with each individual couple for 2 sessions to evaluate their ongoing problems and define the treatment goals, 2) a 2-session educational workshop that provides information about post-deployment strains and mental health sequelae to all veterans and their partners, and 3) twice-monthly multi-couple group meetings for 6 months (12 sessions) that provide a structured format, including out-of-session practice, to build problem-solving, emotion-regulation and communication skills while receiving social support. The multi-group session’s skills training sessions are delivered in three phases.

In phase I (sessions 1–3), the participants are introduced to formal problem-solving methods (i.e., operationalizing the problem, generating solutions non-judgmentally, evaluating the pros and cons of each solution, picking a solution and planning the implementation), using concrete problems in daily living related to PTSD or mTBI (e.g., difficulty remembering scheduled appointments, chores, engaging in family activities in crowded areas) that are generated by the participants. Non-affectively loaded problems are selected initially to facilitate skill acquisition. Group participation is encouraged to foster social support and build a working alliance between the group members and the clinicians towards a common goal. Phase II (sessions 4–6) teaches skills to facilitate accurate recognition, labeling, and regulation of negative emotions that are experienced by the veterans and their partners. In session 4, they members learn mindfulness “what” (i.e., observe, describe and participate) and “how” (i.e., non-judgmentally) skills [70]. These skills help the veterans to learn to or relearn to pause and self-reflect between processing the external stimulus and generating a behavioral response, an important foundation of emotion regulation. Session 5 focuses on crisis survival or distress-tolerance skills (distraction, self-soothing and improving the moment) and acceptance, whereas session 6 focuses on advanced emotion-regulation skills that might be implemented once the acute distress has passed as well as skills to prevent or reduce reactivity to negative emotions in the future, including maintaining healthy eating habits, establishing an exercise routine and practicing good sleep hygiene. Phase III (sessions 7–11) builds on the skills that were learned in phases I and II to increase the awareness of dysfunctional communication patterns and substitute more effective ways of interacting to increase intimacy, marital/relationship satisfaction and the ability to negotiate and effectively solve complex interpersonal problems. It begins with a discussion of “relational mindfulness” [71], which is defined as being mindful of one’s partner’s as well as of one’s own thoughts and feelings.

The communication skills that are taught in MFG-MC (active listening, expressing positive and negative feelings, making a positive request, requesting a time-out, and negotiating and compromising) are drawn from the BFT manual [72] but have been adapted to incorporate emotion-regulation strategies to enhance their effectiveness in this cohort. Couples are told that the skills are composed of specific steps that can be difficult to follow when emotions and/or conflict are high and that it is important to practice emotion-regulation skills to use the skills effectively. For example, the communication skill “expressing negative feelings” in the BFT manual has been reframed as “expressing negative feelings mindfully”. As taught in MFG-MC, this skill begins with a preparation step in which the individual pauses mindfully to examine his/her internal experience and action urges and to consider the impact of expressing negative feelings on the partner/relationship. The questions that are examined during the preparation step include “What is the anticipated outcome on the relationship of expressing negative feelings?”, “Can expressions of negative emotions reinforce our dysfunctional communication patterns?”, and “Can expressions of negative feelings mask underlying feelings that are more potent contributors to my current relationship distress?” This mindful introspection serves as one form of emotion regulation. If the individual decides to proceed with the communication, and the discussion becomes heated, the partners are instructed to request a time-out to avoid dysregulated, reactive responding. When requesting a time-out, the person is instructed to give a reason, rather than simply storming out. For example, the person might state that he/she feels unable to proceed constructively, that his/her emotions are taking over and that it will be better to resume at another time. Participants are also instructed to give a timeframe for resuming the discussion or at least indicate an intention to resume the discussion when “I am able”. During the time-out, each partner is encouraged to practice mindfulness and distress-tolerance skills such as distraction, self-soothing and acceptance to reach a state of mind and affective stability that would permit a constructive discussion. These modest additions to the “expressing a negative feeling” and “time-out” skills that are taught in BFT take into account and acknowledge the potential reactions of the other person and, thus, are practicing relational mindfulness.

#### Efficacy of MFG-MC

The aforementioned RCT is ongoing; however, the initial open trial pilot study with 11 veterans and 14 partners found that the intervention was effective in reducing veterans’ PTSD symptoms (pre–posttreatment Cohen’s *d* = 0.82), anger management (*d* = 0.61), instrumental and subjective social support (*d* = 0.85) and vocational functioning (*d* = 1.03). Participation in MFG-MC was also associated with reduced family burden (*d* = 1.03) and increased family empowerment (*d* = 1.66) [11]. Feedback elicited from the participants in the final session of each group also supported the value of incorporating skills training in ER and communication skills training. As one veteran stated, “… a lot of this stuff I did utilize, like time-outs and stuff … There were times I wanted to fly off the handle. I had to remember some of the things you all taught me”. In the words of a partner, “We needed to know how … to learn to communicate, effective communication, not what we thought communication is … but really understanding what it means to have effective communication”.

## Conclusions

### Limitations and future directions

If they are fortunate, PTSD survivors have the opportunity to recover while living and interacting with important people in their life. The difficulties in the lives of these survivors and their loved ones often result from the impact of PTSD symptoms and comorbidities on marital and family relationships, highlighting the potential importance of conjoint treatments. Tremendous progress has been made in this treatment approach in the last 30 years, moving from simple clinical observations and speculation to rigorously conducted, theoretically rich experimental trials of innovative couples interventions. The initial results of research on both SAT and MFG as well as the findings of research on CBCT for PTSD suggest that embedding PTSD treatment within a relational context might be an effective way to reduce PTSD while also enhancing the couple’s or family’s ability to support veterans’ recovery. In these approaches, relatives learn to help veterans to manage the powerful trauma-related emotions that impact their relationships while also acquiring the communication and problem-solving skills to cope with the stresses and problems in life. Embedding behavioral treatments that provide emotion-regulation skills in a couples therapy (SAT) or multi-couple group (MFG-MC) context has the potential to yield immediate post-treatment reductions in PTSD while also having the promise of improving the family’s capacity to support long-term PTSD recovery. In support of this thesis, a recent review of the role of negative affect in the development of PTSD across multiple trauma populations argued that negative affect disrupts the cognitive processes that are needed to participate in cognitive behavioral therapies fully and recommended ways to incorporate regulating negative affect prior to beginning cognitive behavioral therapy [73].

There are still important research questions to be addressed. They include determining if PTSD couple treatments that incorporate emotion-regulations skills training confer equal benefits as cognitive-behavioral approaches such as those developed by Monson et al. [50]. Additional research should also highlight if specific PTSD symptoms are especially responsive to emotion-regulation strategies or might be differentially responsive to specific ER strategies. Studies of treatment matching might help to determine whether couples who are dealing with dysregulated behavior might benefit more from interventions that incorporate emotional-regulation skills training, whereas veterans who are struggling more with troublesome thoughts (such as those associated with moral injury [74]) might benefit from more cognitive strategies. Most studies to date have focused on PTSD symptom outcomes and relationship satisfaction. Future studies might benefit from the inclusion of a broader range of outcomes, specifically psychosocial re-integration, functional and/or vocational outcomes, to address the important question of whether improvement in symptoms and relationship satisfaction facilitates veterans’ recovery within the community. Future studies of combat veterans should also examine traumatic events preceding military service, as the impact of combat-related trauma might vary in relation to the presence of prior trauma and the veteran’s adaptation. Finally, replication studies are needed.

## Abbreviations

BFT: Behavioral family therapy
CBCT: Cognitive behavioral conjoint therapy
EFT: Emotionally focused therapy
ET: Exposure therapy
HE: Health education
IBCT: Integrative behavioral couple therapy
MFG: Multifamily group
MFG-MC: Multifamily group for military couples
mTBI: Mild traumatic brain injury
OEF/OIF/OND: Operation Enduring Freedom/Operation Iraqi Freedom/Operation New Dawn
PFE: PTSD family education
PTSD: Post-traumatic stress disorder
RCT: Randomized controlled trial
REACH: Reaching Out to Educate and Assist Caring, Healthy Families
SAT: Structured approach therapy
SMI: Serious mental illness
VA: Veterans affairs
